# A Pilot Randomized Controlled Clinical Trial to Test the Feasibility, Acceptability, and Usability of iPREPARED; A Mobile Health Technology for Patients and Care Partners

**DOI:** 10.56392/001c.127872

**Published:** 2025-01-17

**Authors:** Ivan N. Ayala, Hannah Friesen, Fernanda Bellolio, Zayn Boustani, Sandeep Pagali, Stephanie Chambers, Paul Musey, Malaz Boustani, Heidi Lindroth

**Affiliations:** 1 Department of Nursing, Nursing Research Division, Mayo Clinic; 2 Department of Nursing, Mayo Clinic; 3 Department of Emergency Medicine, Mayo Clinic; 4 Health, 3 University of Indiana School of Public Health; 5 Internal Medicine, Mayo Clinic; 6 Emergency Medicine, Indiana University School of Medicine; 7 Aging Research, Health Innovation and Implementation Science, Indiana University School of Medicine

**Keywords:** clinical trial, nonpharmacological, Delirium

## Abstract

**Background:**

Family participation in the delivery of nonpharmacological measures has shown in past studies to prevent 17–75% of incident delirium. A scalable and sustainable method to partner with family (i.e., care partners) and support their delivery of nonpharmacologic measures is needed.

**Objectives:**

To test the feasibility, acceptability, and usability of iPREPARED in a pilot randomized controlled clinical trial (RCT). iPREPARED is a mobile health technology co-designed with patients and care partners containing resources on nonpharmacological interventions to prevent delirium.

**Methods:**

Hospitalized adults (≥65 years) with ≥1 delirium risk factor and a care partner (family or friend ≥18yo) willing to participate were enrolled. The primary outcomes were feasibility, acceptability, and usability. Descriptive statistics are reported.

**Results:**

In total, 23 dyads completed the study (median age 72 years (IQR 64, 79), 60% male, and 100% Caucasian). Dyads reported that iPREPARED was acceptable, with moderate usability. Recruitment in the emergency department was feasible whereas recruitment once hospitalized and admitted was more difficult.

**Conclusion:**

The study design was feasible. iPREPARED was acceptable and usable as an intervention.

## INTRODUCTION

Delirium is an acute brain dysfunction that impacts upwards of 30% of older adults who are admitted to the hospital.^1^ It is independently associated with mortality and future cognitive decline, including dementia.^2^ Multicomponent, nonpharmacological interventions can prevent up to half of incident delirium, however, the adoption of these interventions into clinical care has been limited due to clinician workload and unavailability of resources such as professional coordinators or volunteers.^3,4^ A potential solution to address this limitation, and scale-up nonpharmacologic interventions, is to partner with the patient and their care partner. As reported by a recent systematic review, nonpharmacologic interventions delivered by care partners prevent up to 75% of the incidence of delirium.^5‑7^ To support their involvement, a scalable and sustainable mobile health technology could provide information, tools, strategies, and resources.^8^

**Figure 1.**
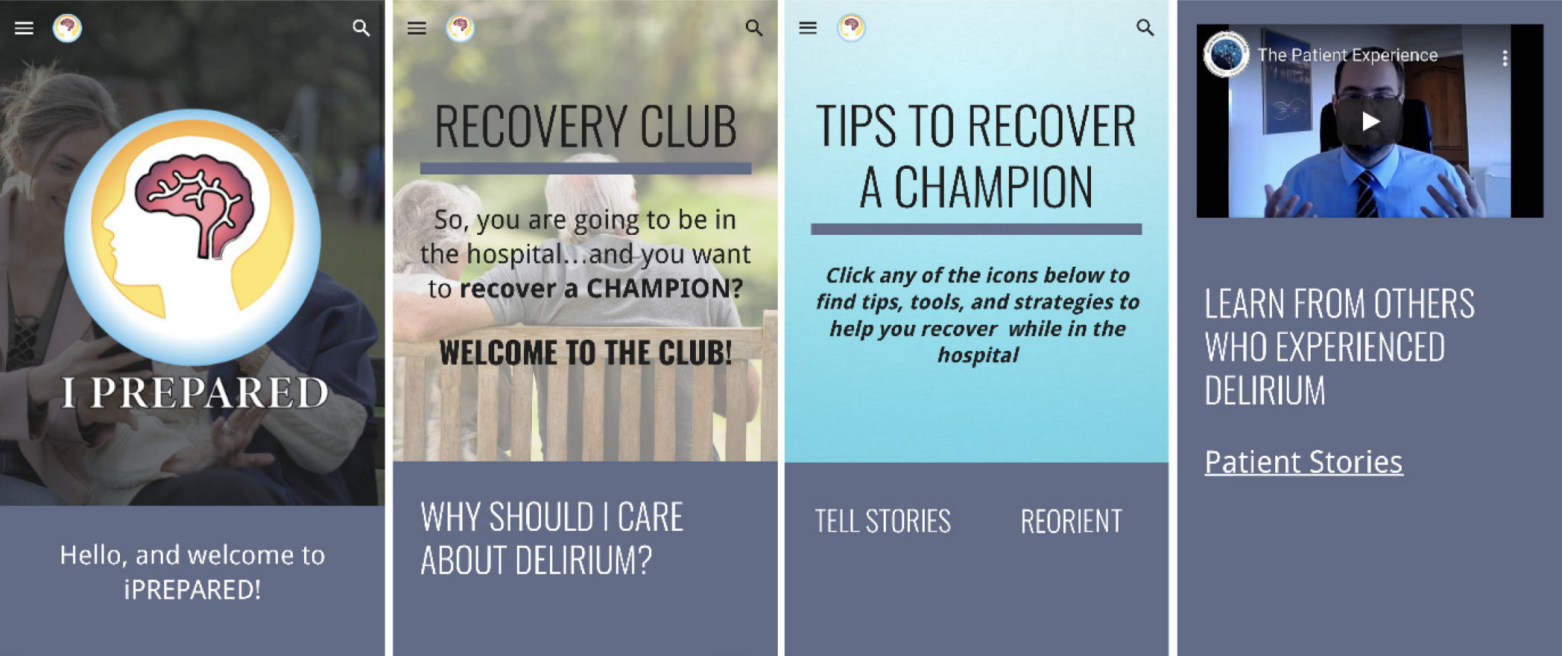
iPREPARED screenshots Figure 1 presents four distinct screenshots depicting the iPREPARED tested prototype (v2.0). These screenshots illustrate various components of the technology interface from left to right. The initial page is first, followed by an introduction on the impact of Delirium. The last two images center on providing practical guidance for recovery and a first-hand experience video of a patient recovered from delirium.

To support care partners in the delivery of nonpharmacologic interventions, a mobile health technology, titled iPREPARED (Figure 1), was co-developed with patients, care partners, and clinicians by applying user-centered design approaches. iPREPARED contains an introductory video, multimedia resources, and tools and strategies to support the delivery of nonpharmacologic interventions to prevent delirium. Multiple stages of usability testing and prototyping were completed. Our primary objective was to evaluate the feasibility, acceptability, and usability of iPREPARED in a prospective randomized controlled clinical trial in hospitalized older adults admitted through the emergency department (ED) and their designated care partner. Secondary objectives included the reduction of delirium severity, delirium-related distress, and qualitative feedback on the usability of iPREPARED. The term “care partner” refers to a spouse, child, caregiver, friend, or neighbor accompanying the patient in the hospital journey. This individual could be paid or non-paid by the patient.

## METHODS

This pilot randomized controlled trial was conducted at a tertiary-level multidisciplinary care hospital in Rochester, Minnesota, which serves both urban and rural populations from the tri-state area. We recruited 30 patient-care partner dyads (n=60 total). Dyads were randomly assigned using computer-generated numbers to the intervention (iPREPARED) or control (usual care) (n=15 patients per group). We used agile methodologies to guide study conduct and intervention development. Institutional Review Board approval was obtained and registered on clinicaltrials.gov (NCT05118867).

## INTERVENTION DEVELOPMENT

To develop iPREPARED, a broad team of transdisciplinary members including patient and care partner representatives, applied user-centered design principles to iteratively build and improve the mobile health technology. Patients and care partners are the target end-users of iPREPARED. Agile Innovation, a theory-based framework, guided the user-centered design process.^9,10^ Each stage aligned with the National Institute of Health (NIH) Model for Behavioral Intervention Development.^11,12^

In Stage 0, the patient experience was investigated using 1:1 semi-structured interviews, journey mapping, and shadowing. Process mapping informed semi-structured question development.^13^ Questions were focused on both the temporal aspects of the patient journey and their lived experience through the process. These interviews were iterative, taking place at different times in the patients journey (e.g., preoperatively, postoperatively, following admission from emergency department) to fully understand all aspects of the patients lived experience. The principal investigator (H.L.) and a user experience design specialist reviewed the interview transcripts and written notes on behavioral observations for insights into areas of opportunity to support the patient in their hospital journey. These insights were shared with the broader team for further discussion and idea generation. Literature reviews were completed to evaluate the current evidence for nonpharmacological interventions to prevent delirium, existing hospital-based programs focused on improving patient outcomes (e.g., the Enhanced Recovery After Surgery program), and theories of behavior change. The study team evaluated each nonpharmacological intervention for its strength of evidence and the potential scalability and sustainability of the intervention following successful adoption into practice. Nonpharmacological interventions that had the highest potential to scale and sustain once adopted, and had strong evidence of efficacy, were selected (reorientation, cognitive stimulation, earplugs/eye mask at night, physical activity, family involvement, addressing sensory deficits). These interventions were then mapped to the Intervention Mapping table developed by Kok et al. (2016) to identify the best methods to target behavior change in patients and care partners.^14^ These are methods that would promote the use of the selected interventions and included: participation, persuasive communication, active learning, tailoring, individualization, modeling, and feedback.

The study team selected the social cognitive theories of the transactional model of stress, self-regulation theory, and behavioral economics to underpin the intervention. Other models and theories, such as the COM-B Model,^15^ Goal-Setting Theory,^16^ and Self-Determination Theory,^17^ were considered during the development process. However, the selected theories were most suitable due to their empirical foundations and specific relevance to the behaviors and psychological processes targeted to mitigate delirium episodes. The theories of self-regulation and the transactional model of stress suggest that individuals develop a mental scheme of how a future event might feel and look like when provided with concrete objective information. Once developed, the mental schema supports the individual when the event occurs. The perception of the event is less stressful, and the person enacts coping strategies to minimize the associated stress. A similar theme emerged from patient interviews; the concept that knowledge of the event prior to the occurrence of the event helped to reduce stress. Explaining what physical sensations the patient might experience following their surgery, including a temporal and environmental-rich description has shown to significantly decrease postoperative delirium, confusion, anxiety, and pain.^18‑25^ Preparatory sensory information has also shown to reduce perceived stress during an ICU hospital stay as well as a decreased ICU and hospital stay.^26,27^ These two theories were combined with behavioral economics. Behavioral economic principles were incorporated throughout iPREPARED using the MINDSPACE tool.^28,29^ Several cognitive biases influence human behavior, either hindering or promoting action or behavior change. The MINDSPACE mnemonic corresponds to the following: We are influenced by who delivers the information (Messenger), what incentives exist to act (Incentives), what others are doing (Norm), the typical route (Default), how important the information is at that time (Salience), if we are ready for the information (Primed), how the information makes us feel (Affect), our agreement to act (Commitment), and how we think of ourselves in relation to the information or action (Ego). Figure 1, displays different screenshots of the tested prototype. Stakeholders (e.g., patients, care partners, clinicians, community members) were asked to rate each iPREPARED prototype based on the MINDSPACE criteria and learnings were incorporated into the next prototype. These interactions took place during 1:1 semistructured interviews, following a similar format as previously outlined.

**Figure 2.**
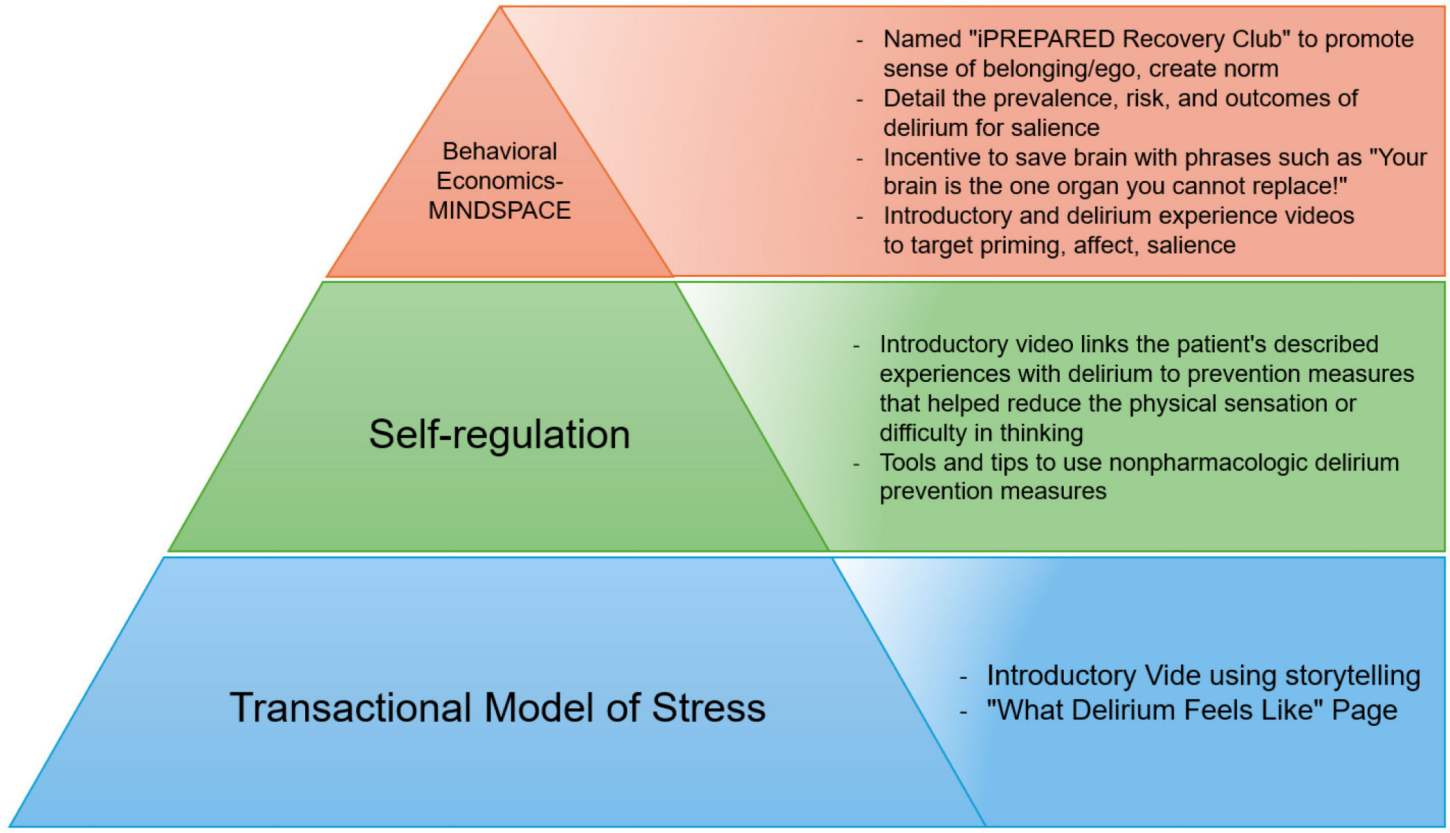
iPREPARED Pyramid Diagram Figure 2 illustrates the theoretical underpinnings of the iPREPARED mobile health technology and link to the corresponding iPREPARED component. This is not an exhaustive list. MINDSPACE Mnemonic. M=Messenger, I=Incentive, N=Norm, D=Default, S=Salience, P=Priming, A=Affect, C=Commitment, E=Ego

These theories, identified evidence, and behavioral change strategies guided the codesign of iPREPARED. This design included an introductory video that represented a “hospital mental schema” and multimedia to promote the delivery of nonpharmacologic delirium interventions to prevent incident delirium. Following the NIH stage model, multiple stages of usability testing and prototype improvement were completed to improve the accessibility, appearance, and multimedia tools available through iPREPARED. Figure 2 illustrates the theories that underpin iPREPARED and connect the theories with the digital components.

In summary, iPREPARED provides the following: 1) an introductory video that provides information in a story-telling format to the dyad on what to expect during their hospital journey and how to apply the protocols provided in iPREPARED; 2) information from delirium survivors on what delirium is and what the patient may experience; 3) delirium risk factors and strategies on how to support the patients during hospitalization; and 4) several tools and strategies on how to use nonpharmacologic delirium interventions including reorientation, sleep hygiene (ear plugs, eye mask), cognitive stimulation through music, reminiscing, gameplay, and others. Additional programming on iPREPARED includes tips from care partners on signs of delirium, what to bring to the hospital to support the patient and complete nonpharmacological delirium interventions, communicating with their healthcare team, and links to delirium-related websites for further support. iPREPARED is built in an adaptable format meaning it can be used on a smartphone, a tablet, or a web browser. To our knowledge, iPREPARED is the first co-designed, theory-driven mobile health technology aimed to support care partners in the delivery of nonpharmacological delirium prevention interventions to reduce incident delirium. The second version of the iPREPARED prototype was tested in this pilot feasibility study.

## ELIGIBILITY

Informed consent was obtained within 24 hours of presentation to the ED or the medical unit. The inclusion criteria were 1) Identified as “intermediate risk” for delirium using the Inouye Delirium Prediction Model^30^; 2) Estimated length of stay >24 hours in the hospital; and 3) have a care partner (>18yo) willing to participate. Patients were excluded if 1) Delirium present in the ED or upon admission to the medical unit; 2) Unable to communicate or participate in the study due to language barriers or sensory deficits (applied to both dyad members); 3) Person who is incarcerated; 4) Patients admitted to hospice service, and; 5) A diagnosis of dementia or scoring <18 on the Montreal Cognitive Assessment (MoCA).^31^ Patients with dementia were excluded from this pilot study as it was important to get patient feedback on the acceptability and usability of the technology. Future trials will include individuals living with dementia and other cognitive impairments. The study team reviewed dashboards reporting on the numbers screened, approached, and enrolled weekly to proactively identify barriers to progress.

## PHASES OF RECRUITMENT

The pilot study underwent two phases of recruitment and consent. This process was guided by agile methodology and the use of a weekly study dashboard. The first phase (12/2021–06/2022) employed division study coordinators (3.22 Full Time Equivalents, FTE) to screen patients using an electronic health record (EHR) generated report. To show up on the report, the patient had to be physically present and admitted on the hospital unit. Coordinators were physically located in an office building, approximately 1 mile from the hospital. They would run the EHR report daily and approach eligible patients during business hours (8:00 a.m. to 5:00 p.m).

The second phase (07/2022–08/2022) employed two study coordinators (.14 FTE) to screen patients in the emergency department (ED) using a central patient tracking board that updated in realtime to display new patients. The ED study coordinators were in the ED and covered evening hours (5:00–8:00 p.m.). Patients who met inclusion criteria (≥60yo, risk for delirium, and had an admission order) were approached for the study and screened out if exclusion criteria were met. Once enrolled, the ED coordinators handed off the participant to the inpatient study coordinator to complete enrollment assessments.

The study dashboard tracked how many patients were screened, categorized as eligible, reasons eligibility was not met, approached, reasons not approached, enrolled, failed cognitive or COVID screenings, declined the study, and reasons withdrawn/withdrew.

## PRIMARY OUTCOMES

The primary outcomes were feasibility of study enrollment, the acceptability of the intervention to both patients and care-partners, and the usability of iPREPARED. Feasibility was measured by the percentage of patients eligible and approached for the study and were enrolled into the trial. The number of patients consented per staffed coordinator hour was calculated. Acceptability was measured using the 4-question, Likert scale (0–4), Treatment, Acceptability, and Preference Questionnaire (TAPQ). The intervention group completed the TAPQ after initially interacting with the technology upon enrollment (pre) and upon completion of the study (post) on hospital day 4 or discharge. The 10-question, Likert scale (1–5), System Usability Scale (SUS) was used to assess usability and was completed upon day 4 or discharge.^32^

## SECONDARY OUTCOMES

Secondary outcomes included delirium incidence (3D-Confusion Assessment Method^33^), severity (Delirium Rating Scale-R-98^34^), and delirium-related distress^35^ and were assessed by trained study team members twice daily until hospital day 4 or discharge, whichever occurred first. Qualitative semi-structured interviews were conducted with the patient and care partner, from the intervention arm, upon study completion to explore usability.

## STATISTICAL ANALYSIS

Demographic and clinical data were summarized using descriptive statistics using IBM SPSS Statistics for Windows, version 28 (IBM Corp., Armonk, N.Y., USA). Feasibility was evaluated by the percentage of participants who consented and completed the study. For eligible patients approached for the study, if >30% consented, and >80% completed the study, it was deemed feasible. The intervention was deemed acceptable if a mean score >3.0 was obtained from the TAPQ (0–4 scale). The intervention was deemed usable if a mean score > 70 was obtained from the SUS (0–100 scale). The preliminary efficacy of the differences between treatment groups and delirium incidence (yes/no) was not examined due to the low number of incident delirium (n=1). The difference in the twice daily total delirium severity score (secondary outcome, all scores regardless of 3DCAM delirium status) and patient-reported delirium-related distress was examined between groups using the Wilcoxon Rank Sum Test, a nonparametric statistical procedure that accommodates smaller sample sizes and non-normality in data distribution. A linear effects model was used to examine the differences between study groups in the Total DRS score across assessments days.

The interviews were transcribed and analyzed for feedback on usability of iPREPARED. A formal qualitative analysis was not completed as the interviews were designed to gather specific feedback on the usability of the mobile health technology. Instead, as we analyzed the feedback, we categorized quotes into specific usability components. These categories and quotes are reported.

## RESULTS

**Figure 3.**
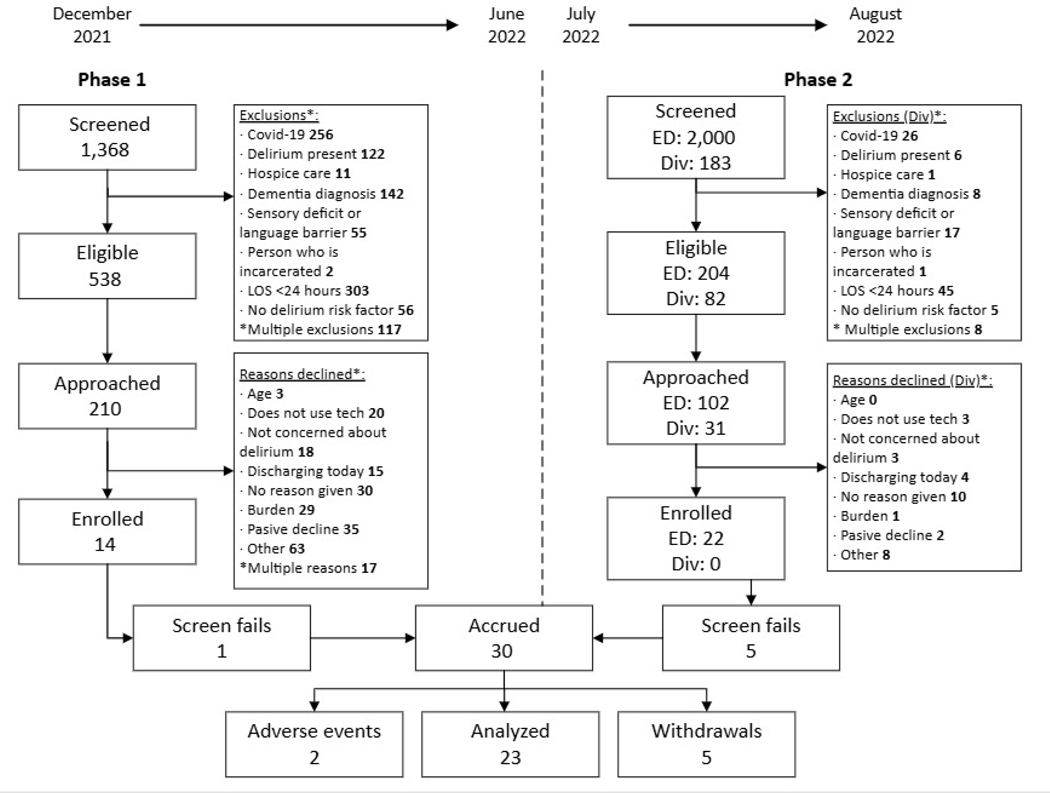
Consort Diagram Figure 3 illustrates the progression of patient enrollment. The study was divided in two phases, phase 1 was led by Division (Div) study coordinators while phase 2 added the efforts of study coordinators located in the Emergency Department (ED). The ED study coordinators followed a different process for screening and approach. Therefore, the numbers screened and approached for the ED in phase 2 are approximate estimates based on monthly admissions to the ED that met baseline study eligibility. The gap between Eligible and Approached patients was due to limited staff availability (50%), timing of the screening report (~30%), and availability of patient (~20%). Patients were categorized as “Screen Fail” if no care partner was available, scored a MOCA of 18 or less, or tested positive for COVID-19 after enrollment. Abbreviations: ED: Emergency Department; Div: Division of Nursing Research

Overall, thirty-six patient-care partner dyads were consented, 6 did not pass cognitive or COVID screening once enrolled. Thirty dyads were enrolled into the study. Seven participants (patient part of the dyad) withdrew from the study due to loss of follow-up (2), did not want frequent study visits (1), and felt overwhelmed (2) and 2 patients experienced adverse events that prevented study completion (1-died, 1-stroke) for a final sample size of 23 dyads. Figure 3 overviews the CONSORT diagram.

The median age was 72 (IQR 64, 79), 60.0% male (n=18), and 100% (n=23) identified as Caucasian and self-reported as not Hispanic or Latino. Table 1 outlines all descriptive demographic and clinical characteristics. Table 2 outlines Primary and Secondary characteristics.

## PRIMARY OUTCOMES

### Feasibility.

The pilot study underwent two phases of recruitment and consent. The monthly recruitment rate in phase 1 ranged from 3–29%. An average of 0.03 patients were enrolled per hour. Reasons for study decline ranged from being too overwhelmed with the clinical care schedule, not concerned about delirium, or believing they would discharge soon. These reasons are outlined in Figure 3.

The monthly recruitment rate in phase 2 ranged from 26–34%. An average of 0.83 patients were enrolled per hour. In the ED, the main reason for study decline was the use of technology. Eighty-two percent of enrolled participants completed the study, indicating low attrition and overall feasibility of the study design with >30% enrolled in the second phase of recruitment.

### Acceptability:

Patients and care partners rated the iPREPARED mobile health technology as acceptable. After interacting with the technology (pre), patients (n=8) rated iPREPARED as acceptable with a median TAPQ score of 2.9 (IQR 2.4, 3.4). Upon completion of the study (n=7), patients maintained a rating score of acceptable, with a median TAPQ of 3.0 (IQR 2.9, 3.4). Similar acceptability ratings were reported by care partners, with a pre median TAPQ of 3.0 (IQR 2.0, 3.6) and a post median TAPQ of 3.0 (3.0, 4.0).

### Usability:

Marginal usability was reported by the patient-care partner dyad (n=6). Patients (n=6) reported the usability of the technology as above average with a median SUS score of 77.5 (50.6–91.5). Care partners (n=3) reported a median SUS of 55.0 (47.5–87.5) indicating below average usability.

## SECONDARY OUTCOMES

### DELIRIUM INCIDENCE

The effect size was not calculated due to the low incidence of delirium (n=1, control group) in the final dataset. Two participants that were withdrawn from the study due to adverse events did experience incident delirium. These are not included in the final dataset due to their withdrawal, however, are described as follows: One participant randomized to the intervention group scored positive on the Day 1 3D-CAM AM assessment. They were transferred to the ICU the next day and the care partner withdrew consent prior to their death. Another participant randomized to the control group was transferred to the neurologic intensive care unit due to an acute on chronic subdural hematoma. They scored positive on Day 1 3D-CAM PM assessment and were later withdrawn from the study due to family request.

### DELIRIUM SEVERITY

**Figure 4:**
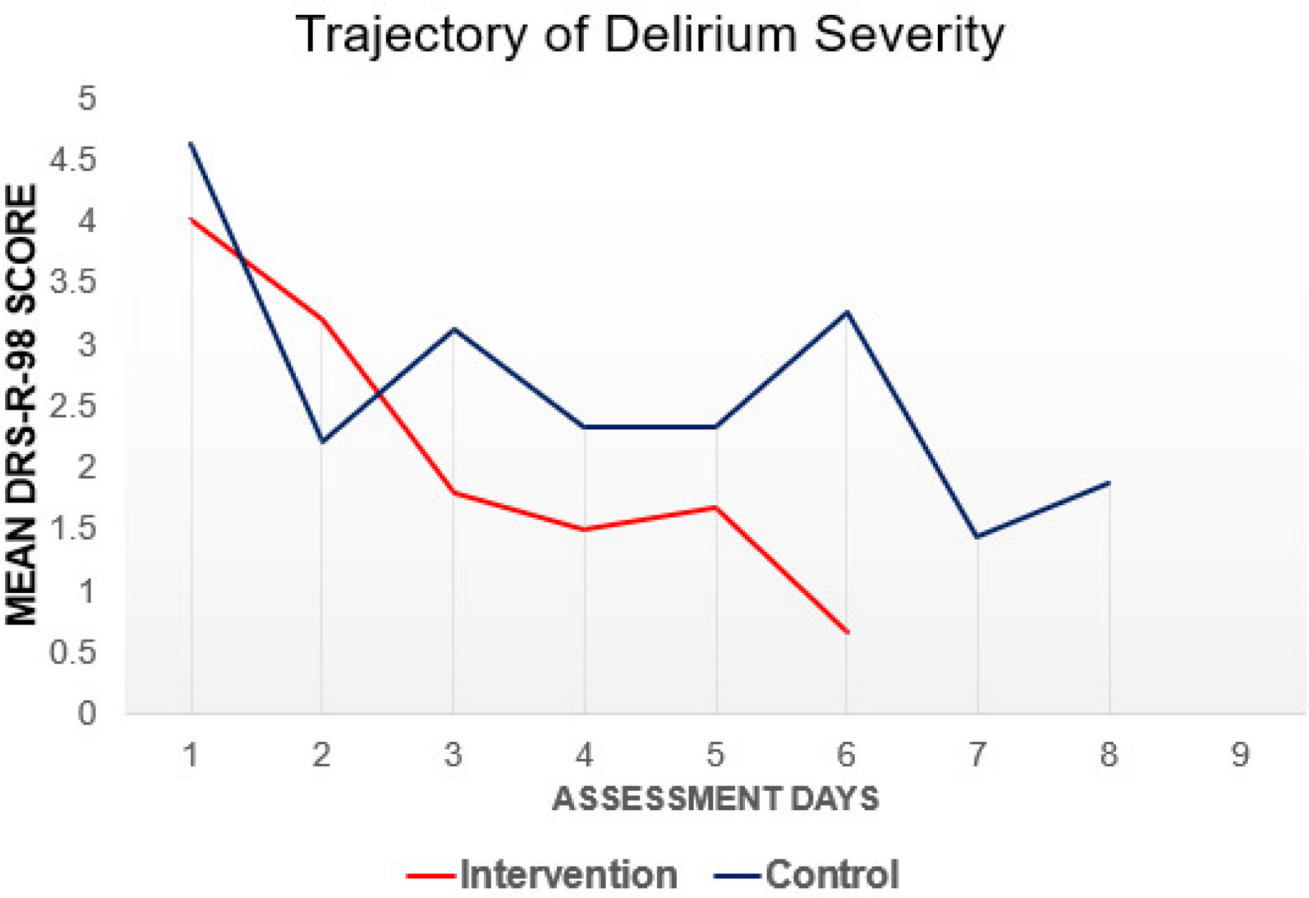
Figure 4 displays the secondary outcome of delirium severity across the number of assessment days. Average DRS-R-98 scores were used to map out the Average Scores (y axis) over the assessment days (x axis). The linear effects model demonstrated a trend towards statistical significance with a faster resolution of delirium severity in the intervention group.

No statistically significant differences were observed between study groups for the total DRS-R-98 score (Intervention: 4.13+2.8 vs Control: 4.47+3.54). A trend towards lower DRS-R-98 total scores was observed in the intervention group, see Figure 4, however the linear mixed-effects model was not significant following Bonferroni correction (p=.36).

### DELIRIUM-RELATED DISTRESS

No statistically significant differences were observed between study groups for patient-reported delirium distress. There is a trend towards lower delirium-related distress scores in the intervention group (Intervention: 1.63±2.00 vs Control: 3.27±3.43, p=.441).

## QUALITATIVE RESULTS

Patient-care partner dyads provided qualitative feedback during semi-structured interviews. The interviews were pragmatic and focused to improve the website. Feedback shared was used to build iPREPARED prototype v3.0. Qualitative data was not sufficient for a rigorous, traditional thematic analysis. However, for readers knowledge, responses were categorized, and representative quotes have been added under each theme in Table 3.

## DISCUSSION

This pilot randomized controlled clinical trial demonstrated adequate feasibility and acceptability of the iPREPARED intervention by the patient-care partner dyad. Enrolling patients in the ED was more successful than enrolling participants once they were admitted to the medical unit. The difference in rates of enrollment shown between study phases is likely due to the differing research processes when screening and approaching patients. Several dyads reported being too overwhelmed and declined participation once admitted to the medical unit. The usability of the intervention was moderate to low and important themes were identified in the qualitative interviews. These learnings were applied to the next iteration of the iPREPARED prototype mobile health technology with improved usability.^36^

The ED setting provides an opportunity to start non-pharmacological interventions early in the course of hospitalization. Delaying the start of delirium prevention protocols was associated with a greater risk of delirium onset in the proceeding 72 hours of hospitalization in one study.^37^ Previous studies demonstrated that care partners facilitated nonpharmacologic delirium prevention measures such as reorientation, cognitive stimulation, and encouragement of mobility/sleep hygiene reduced the incidence of delirium by 17–75%.^5,6^ The Geriatric Emergency care Applied Research Network (GEAR) highlighted the need for best practices in delirium prevention, recognition, and treatment, and emphasized the importance of patient and care partner involvement.^38^ Next steps will be to develop a larger clinical trial focused on scalability of iPREPARED and the clinical outcomes of these nonpharmacological interventions.

This pilot RCT had a lower delirium incidence rate than expected. The incidence delirium rate was expected to be 12%.^38‑40^ The lower-than-expected incidence rate could be attributed to the first phase of recruitment, which approached patients already admitted to their hospital room. It was common for patients to decline participation when they had several procedures and tests scheduled. These patients likely had a higher acuity of illness. This observation is one of the reasons recruitment was modified in phase 2 to focus on patients in the emergency room. At this point in their care journey, patients were not aware of future procedures and tests. Their main reason for declining participation was the use of technology (phase 2) versus feeling overwhelmed and burdened (phase 1). Lastly, two patients (1-control, 1-intervention) that did have incident delirium withdrew from the study and are not included in the final analysis. These patients experienced significant adverse events during their hospital stay and were withdrawn by their care partners. If these patients had remained in the study, the incident delirium rate would be closer to the estimated rate. Future studies that are focused on this population of patients should consider enrolling in the ED as it was more feasible compared to once patients were admitted.

## LIMITATIONS AND STRENGTHS

This pilot study was primarily descriptive and lacked wider representation from culturally and linguistically diverse communities. Relationships between variables are not explorable due to the small sample size and low delirium incidence. The strengths of the study include a well-trained study team that has expertise in delirium, user-centered design, and agile principles. Future studies should further test iPREPARED in a larger sample size with a diverse cohort of patients.

## CONCLUSION

In a pilot randomized clinical trial that recruited aging adults admitted to the hospital through the emergency department (ED) with an intermediate risk for delirium, and their designated care partner, found that study recruitment was feasible when recruitment was completed in the ED. Participants rated the intervention, iPREPARED, as acceptable. Feedback on the usability of the mobile health technology was used to improve the next prototype. Future research and larger scale studies should consider recruiting patients in the ED setting, before they transfer to their hospital room as eligible patients quickly became overwhelmed once admitted. iPREPARED is a scalable and sustainable mobile health technology that has great potential in supporting care partners to reduce incident delirium. The effectiveness of this approach needs to be tested in a larger scale RCT.

## Figures and Tables

**Table 1.** Overview of descriptive statistics

Variables	Total N=30	Intervention N=8	Control N=15	Withdrawals N=7

***Age*** *(median, IQR)*	71 (65, 79)	71 (70, 74*)*	*73 (67–79)*	*6*5 (63, 82)
***Female***, *n(%)*	12 (40)	3 (37.5)	5 (33.3)	4 (57.1)
***Ethnic background***				
*Caucasian*	30 (100)	8 (100)	16 (100)	7 (100)
*Non-Hispanic*	30 (100)	8 (100)	16 (100)	7 (100)
***Education level***				
*12*	8 (26.7)	2 (25)	3 (20.0)	3 (42.9)
*>12*	20 (66.7)	6 (75)	11 (73.3)	3 (42.9)
*Missing*	2 (6.7)	0 (00)	1 (6.7)	1 (14.2)
***Occupational Status***				
*Retired*	22 (73.3)	6 (25)	10 (66.7)	6 (85.7)
*Part-time employment*	1 (3.3)	2 (75)	1 (6.7)	0 (0)
*Full-time employment*	5 (16.7)	0 (0)	2 (13.3)	1 (14.3)
*Unknown*	1 (3.3)	0 (0)	1 (6.7)	0 (0)
*Missing*	1 (3.3)	0 (0)	1 (6.7)	0 (0)
***MoCA Total Score*** *Median (IQR)*	23.0 (22.0, 26.0)	26.0 (22.3, 27.0)	22.5 (21.8, 26.0)	23.0 (22.0, 24.0)

Table 1 summarizes the descriptive demographic and clinical variables.

Abbreviations: MoCA: Montreal Cognitive Assessment.

**Table 2.** Overview of Outcomes

Variables	Total N=30	Intervention N=8	Control N=15	Withdrawals N=7

***Primary Outcome – Acceptability (TAPQ),*** *Median (IQR)*				
***Baseline Patient***		2.9 (2.1, 3.6) N=8		3.0 (2.8, 3.5) N=5
***Baseline Care Partner***		3.0 (1.1, 3.9) N=4		3.0 (2.5, 3.5) N=2
***Discharge Patient***		3.0 (2.8, 3.5) N=7		
***Discharge Caregiver***		3.0 (1.8, 4.0) N=5		
***Primary Outcome – Usability (SUS score),*** *Median (IQR)*				
***Discharge, Patient (n=6)***		77.5 (50.6, 91.5)		
***Discharge,*** ***Care Partner (N=3)***		55.0 (47.5, 87.5)		
***Secondary Outcome - Delirium (3D-CAM)***, *n(%)*				
*Yes*	1 (3.3)	0 (0)	1 (6.7)	2 (28.6)
*No*	29 (96.7)	8 (100.0)	14 (93.3l)	5 (71.4)
***Secondary Outcome – Delirium severity, Delirium-related distress,*** *Mean (SD)*				
*DRS-R-98, peak*	5.17 (4.62)	4.13 (2.80)	4.47 (3.54)	7.86 (7.31)
*Distress, peak*	3.06 (3.07)	1.63 (2.00)	3.27 (3.43)	4.33 (3.01)

Table 2 summarizes the primary and secondary outcome variables. The Wilcoxin Rank Sum Test was used to examine the difference between groups on the DRS-R-98 (p=.966) and delirium-related distress (p=.441)

Abbreviations: 3D-CAM: 3D-Confusion Assessment Method; DRS-R-98: Delirium Rating Scale; TAPQ: Treatment Acceptability and Preference Questionnaire; SUS: System Usability Score

**Table 3.** Qualitative Interview Quotes per Usability Category

**Category 1:** **Acceptance and use of the tool**		
Interviewee	Quote	Supportive vs Critical
Patient	*“[I am] very comfortable using it, didn’t feel lost. Very comfortable with it.”*	Supportive
Care Partner	*“I think it is very thorough and useful.”*	Supportive
Care Partner	*“[I] haven’t seen the website. [Uses social media more].”*	Critical
Care Partner	*“I did not use it. I did not see it. I did not access it. I am not sure if I can answer any of these questions.”*	Critical
**Category 2:** **Need for further development specifically regarding language and videos**		
Interviewee	Quote	Supportive vs Critical
Patient	*“The language needs to be more direct and clear on the website.”*	Critical
Care Partner	*“I thought it was childish. I felt talked down to, it was too basic.”*	Critical
Patient	*“It seemed to be fine…. I think it needs a little more development, but otherwise I think it seems fine.”*	Supportive
Care Partner	*“[Need to incorporate] real people that were more formal, more official, more serious, giving real examples.”*	Critical
Care Partner	*“The video could contain the same type of information but in a different format. Real descriptions of things you are looking for in delirium.”*	Critical
**Category 3:** **Increased patient awareness of delirium in self or others**		
Interviewee	Quote	Supportive vs Critical
Patient	*“I just didn’t know what it was all about until I read the information. Then I was very impressed because I never thought about delirium. I felt so bad for the people with confusion.”*	Supportive
Patient	*“I just started it last night and I’ve done like.. what is delirium? and .. um.. oh there were a couple other little pages that I started reading through last night. Well.. it’s it’s interesting (iPREPARED)… yeah I kind of enjoyed.. like I said.. when I was in Madison, they had this one picture on my wall.. and it was .. It was like a French door and I mean it was like the flowers and all open and every once in a while… it was like you could see my dad peaking over it… (haha) he’s been dead for 6 years.. (haha) yeah.”*	Supportive
Patient	*“kind of put those things together.. correlate those things.. and I mean there was one medicine when I was in here.. they gave me for something.. and it was like ..they ask me how.. and I said “ well you know what my mom has been dead for 30 years.. but I said we have had the nicest conversation this afternoon…”*	Supportive
Patient	*“yes… yes I think it does [help]… because I’ve seen family members you know with it.. and and ..and they were afraid and didn’t know what was going on and ..you know.. they didn’t.. they just were out of sorts and weren’t their selves at all. And I think maybe if they understood it a little bit and even talking to them.. I mean myself talking to them .. I think if it was presented the way you people do it.. They probably would have understood what was happening better.”*	Supportive
**Category 4:** **Patient use of delirium prevention strategies**		
Interviewee	Quote	Supportive vs Critical
Patient	*“Umm.. yeah during a couple points in my stay.. I um ..to get through the pain.. there was some deep breathing and then umm.. trying to sleep.. closing the door, turn the lights off turning… make it quiet. I know they suggest music and a couple other thing… I am kind of the opposite I like things quiet and black. That’s how I relax”*	Supportive
Patient	*“Nope haven’t used any of those yet (delirium prevention strategies)”*	Critical
Patient	*“I did some deep breathing periodically but other than that, that’s about it.”*	Supportive
